# Frequency of Atrial Fibrillation in Patients Presenting With Decompensated Heart Failure

**DOI:** 10.7759/cureus.20594

**Published:** 2021-12-22

**Authors:** Rafi Ullah, Ahmad Shiraz, Sher Bahadur, Farhat Shireen

**Affiliations:** 1 Cardiology, Lady Reading Hospital Peshawar, Peshawar, PAK; 2 General Surgery, Hayatabad Medical Complex Peshawar, Peshawar, PAK; 3 Epidemiology and Public Health, Khyber Institute of Child Health, Peshawar, PAK

**Keywords:** comorbidities, risk factors, heart failure, hypertension, atrial fibrillation

## Abstract

Background

Atrial fibrillation (AF) is a common concern in patients with heart disease, especially those with acute decompensated heart failure (ADHF). We conducted a cross-sectional study to determine the frequency of AF and associated risk factors among patients with ADHF at a tertiary care hospital in Peshawar, Pakistan.

Methods

We conducted a cross-sectional analytical study of hospitalized patients with ADHF treated in a tertiary care hospital in Peshawar, Pakistan, from June 5 to October 30, 2021. The study's primary outcome was the proportion of patients with ADHF who had AF, and our secondary outcome was examining the risk factors for AF. The College of Physicians and Surgeons Pakistan provided ethical approval of the study design. Data were analyzed using IBM SPSS Statistics for Windows version 24.0 (IBM Corp., Armonk, NY, USA). We applied the chi-square test to compare the proportion of AF concerning risk factors (i.e., comorbidities).

Results

One hundred ninety-four patients with ADHF were included in the study; 54.6% were male and 45.4% female. Most (56.7%) were older than 60, and 38.1% were aged 40-60. The prevalence of AF was 38.1%. Diabetes, hypertension, previous stroke, myocardial infarction (MI), and chronic obstructive pulmonary disease (COPD) were the most common comorbidities. All patients with ADHF with AF also had MI and hypertension. Patients of known coronary artery disease (CAD) but without MI, previous percutaneous coronary intervention (PCI), or coronary artery bypass graft (CABG) surgery were less associated with AF than other comorbidities.

Conclusions

We conducted this study to determine the incidence of AF among patients with ADHF. AF occurs in a significant amount of patients with ADHF, and the risk factors associated with AF in these patients include hypertension, history of MI, diabetes, and COPD. Healthcare professionals should screen patients with ADHF for AF, especially those with common risk factors.

## Introduction

Atrial fibrillation (AF) is a common concern for patients with cardiac disease, especially those with arrhythmia and acute decompensated heart failure (ADHF) [1,2]. Approximately 20%-35% of admitted patients with ADHF have AF, even in the early stage of the disease [3]. While a high frequency of AF concurrent with ADHF has been reported, significant regional differences exist. In Pakistan, this issue has not been prioritized in previous research. The risk factors and adverse effects of AF in patients with heart failure vary from person to person and region to region because of differences in lifestyle, comorbidities, healthcare facilities, and treatment compliance. Therefore, we conducted this study to determine the frequency of AF and associated risk factors among patients with ADHF at a tertiary care hospital in Peshawar, Pakistan.

## Materials and methods

This hospital-based, cross-sectional study was conducted in a public sector teaching hospital in Peshawar, Pakistan, from June 5 to October 30, 2021, using consecutive sampling. Ethical approval was granted by the College of Physicians and Surgeons Pakistan on June 4, 2021. The study included men and women older than age 18 who presented with ADHF to the hospital as confirmed by echocardiogram. We excluded patients with a history of rheumatic heart disease and congenital abnormalities (also confirmed by echocardiogram). All study participants provided written informed consent to be included. All patients with ADHF were assessed for the presence of AF based on an electrocardiogram. We obtained detailed clinical history for known comorbidities. We also evaluated previous investigations and prescriptions for all participants. According to the official data, approximately 600 patients of ADHF presented annually, so assuming 20% anticipated population [3], 0.05% precision, and 95% confidence level and considering 10% loss to follow-up, the expected sample size was 194.

We collected data using a structured proforma, transferred the information into a Microsoft Excel spreadsheet (Microsoft Inc., Redmond, WA, USA), and then analyzed using IBM SPSS Statistics for Windows version 24.0 (IBM Corp., Armonk, NY, USA). Descriptive statistics are presented for the proportions of comorbidities and coexistence of AF. We used the chi-square test to determine the associations between the risk factors for AF in patients with ADHF for comparative analysis. P-values less than 0.05 were considered statistically significant.

## Results

**Figure 1:**
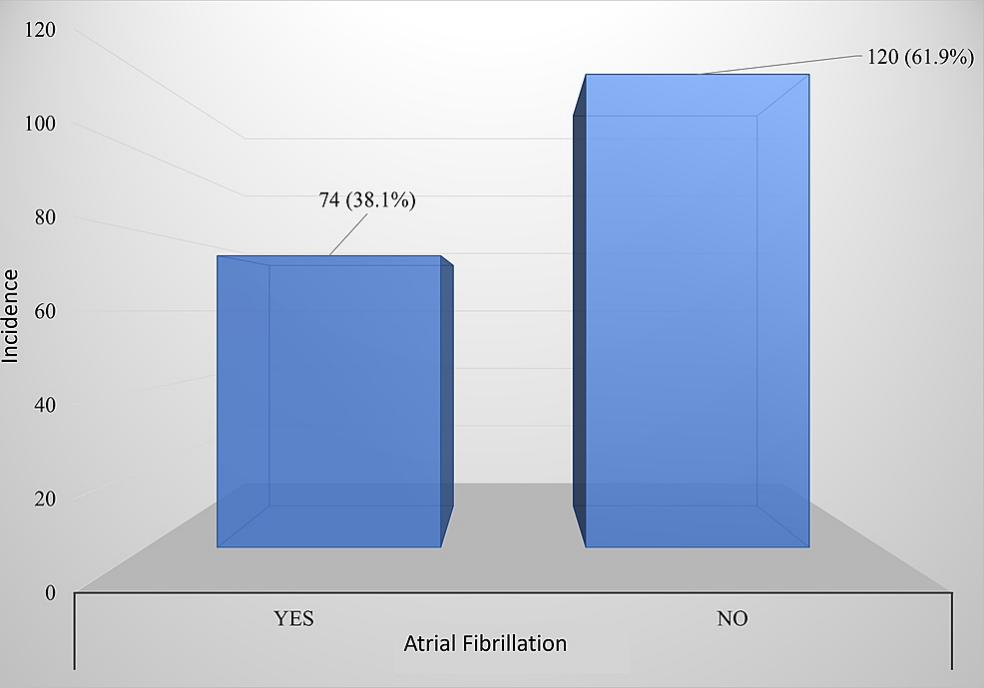
Frequency of AF among patients with ADHF AF: atrial fibrillation; ADHF: acute decompensated heart failure

A total of 194 patients were included in the study (106 men (54.6%) and 88 women (45.4%)). Most patients (n = 110 (56.7%)) were older than age 60, followed by aged 74 (38.1%), aged 40-60 (10%),and then aged 20-40 (5.2%). AF was found in 38.1% of patients with ADHF (Figure 1).

In our study, the most common comorbidities among patients with ADHF were myocardial infarction (MI) (n = 148 (76.3%)), hypertension (n = 101 (52.1%)), diabetes (n = 98 (50.5%)), chronic obstructive pulmonary disease (COPD) (n = 89 (45.9%)), coronary artery disease (CAD) (n = 86 (44.3%)), stroke (n = 82 (42.3%)), previous coronary artery bypass graft (CABG) (n = 25 (12.9%)), and percutaneous coronary intervention (PCI) (n = 24 (12.4%)) (Table 1).

**Table 1:** Common comorbidities associated with ADHF ADHF: acute decompensated heart failure; CAD: coronary artery disease; MI: myocardial infarction; PCI: percutaneous coronary intervention; CABG: coronary artery bypass graft; COPD: chronic obstructive pulmonary disease; TIA: transient ischemic attack

Parameters	Status			
	Yes	f(%)	No	f(%)
CAD	86	(44.3)	108	(55.7)
History of MI	148	(76.3)	46	(23.7)
Previous PCI	24	(12.4)	170	(87.6)
Previous CABG	25	(12.9)	169	(87.1)
Diabetes	98	(50.5)	96	(49.5)
COPD	89	(45.9)	105	(54.1)
Hypertension	101	(52.1)	93	(47.9)
Previous stroke/TIA	82	(42.3)	112	(57.7)

Patients with concurrent AF and ADHF were significantly positive for a history of MI, CAD, diabetes, hypertension, and previous stroke/transient ischemic attack (TIA), but sex had no significant association with AF concurrent with ADHF (p = 0.49). In patients with AF, 57 (77%) had a history of CAD, 66 (89.2%) were positive for a history of MI, and 49 (66.2%) had diabetes. Hypertension was the leading comorbidity (n = 53 (71.6%)), followed by COPD (n = 48 (64.9%)), and previous stroke/TIA (n = 44 (59.5%)). Only 14 (18.9%) patients had a history of PCI, and 18 (24.3%) had previous CABG. The differences for all these parameters were significant (p < 0.001) (Table 2).

**Table 2:** Comparison of risk factors among patients ADHF with and without AF ADHF: acute decompensated heart failure; AF: atrial fibrillation; CAD: coronary artery disease; MI: myocardial infarction; PCI: percutaneous coronary intervention; CABG: coronary artery bypass graft; COPD: chronic obstructive pulmonary disease; TIA: transient ischemic attack

Parameters/comorbidities		Atrial fibrillation		
		ADHF with AF (n = 74)	ADHF without AF (n = 120)	P-value
Gender	Male	45 (60.8%)	67 (55.8%)	0.49
	Female	29 (39.2%)	53 (44.3%)	
CAD	Yes	57 (77%)	29 (24.2%)	0.0001
	No	17 (23%)	91 (75.8%)	
History of MI	Yes	66 (89.2%)	82 (68.3%)	0.001
	No	8 (10.8%)	38 (31.7%)	
Previous PCI	Yes	14 (18.9%)	10 (8.3%)	0.03
	No	60 (81.1%)	110 (91.7%)	
Previous CABG	Yes	18 (24.3%)	7 (5.8%)	0.0001
	No	56 (75.7%)	113 (94.2%)	
Diabetes mellitus	Yes	49 (66.2%)	49 (40.8%)	0.001
	No	25 (33.8%)	71 (59.2%)	
COPD	Yes	48 (64.9%)	41 (34.2%)	0.0001
	No	26 (35.1%)	79 (65.8%)	
Hypertension	Yes	53 (71.6%)	48 (40%)	0.0001
	No	21 (28.4%)	72 (60%)	
Previous stroke/TIA	Yes	44 (59.5%)	38 (31.7%)	0.0001
	No	30 (40.5%)	82 (68.3%)	

## Discussion

AF in patients with ADHF is relatively common and has a poor prognosis, but its prevalence and risk factors seem to vary by country [4]. Therefore, this study sought to determine the frequency of AF among patients with ADHF in Pakistan. Our results indicate that the prevalence of AF among patients with ADHF was 38.1%, which is supported by data from Turkey, where 39% of patients with ADHF had a history of AF [5]. However, 25.4% of the subjects had AF in Cameroon [6]. In Brazil, a review of five years of hospital records found that 40% of patients with ADHF had AF [3]. In another study, approximately 20%-35% of patients diagnosed with ADHF had AF at the time of admission, and one in three cases of AF were acute [7].

Also, AF was positively associated with advanced age (26% for men and 23% for women older than age 40 [8]), ischemia, right ventricular (RV) dysfunctions, high ejection fraction (EF), and cardiomegaly, especially an enlarged left atrium (p < 0.05) [1,3]. Additional risk factors reported by the literature include a history of hypertension, diabetes, MI, medications, socioeconomic status, and body mass index [9,10].

AF can lead to decompensation, which may act as a primary stimulus to acute heart failure. Because of this decompensation, there is an increase in left atrial pressure and decreased stroke volume. Patients with AF and ADHF frequently present with mitral valve regurgitation and have a poor prognosis with a high risk of stroke and increased risk of death [11,12]. Mortality was high for patients hospitalized more than once for AF and ADHF [13]. According to the literature, 39% of patients with ADHF have a positive history of AF and were often in advanced age [1,2,5]. AF in ADHF was associated with stroke, increased blood pressure, and valvular diseases [5]. These changes make AF resistant to treatment using rhythm-controlled strategies [14]. Another study in Pakistan regarding the risk factors for AF reported that 55.3% of patients had a history of rheumatic heart disease, 34% had ischemia, 28% presented with RV dysfunction, and 34.5% had mitral valve regurgitation [10].

Heart failure incidence is associated with the time of heart failure onset. One study reported that at the acute stage, the incidence of AF is 10.3%, and the incidence rate was 27.3/1000 person-years [15]. A 75% increase in the occurrence of AF takes place in the first six months of heart failure (odds ratio: 3.6). This suggests that AF is high in the initial phase of HF, particularly within six months of onset [15].

MI was the most common comorbidity in our study population (77.3%), followed by hypertension (52.1%) and diabetes (50.5%). CAD (44.3%) and stroke (42.3%) were also present, but only a few instances of CABG (12.9%) and PCI (12.4%) were noted. Our findings were consistent with other reports where CAD was the second leading comorbidity after hypertension among patients with ADHF. One study reported that hypertension (70%) and CAD (60%) were present in patients with ADHF, followed by valvular problems (44%), diabetes (40%), cardiomyopathy (25%), and renal impairment (20%) [16]. Another study reported a positive association between AF and advanced age (p < 0.0001) [3]. In that study, AF was also common in patients with RV dysfunction, left atrium enlargement, hypertension, low EF, and nonischemic heart disease. AF was also associated with increased hospital stay and high in-hospital mortality [3].

Comorbidities such as MI, hypertension, CAD, diabetes, and COPD were strongly associated with AF (p < 0.001). However, AF was less common among patients with a previous history of stroke, CABG, or PCI. Other studies have confirmed that these comorbidities are associated with adverse clinical outcomes due to atrial stiffness, vasoconstrictions, and inflammation [17,18]. Kazeminia et al. reported that diabetes was the most common comorbidity among patients with heart failure, and the prevalence increases over time [19]. Diabetes is associated with half of the mortality among patients with ADHF [20]. Joseph et al. found that 70% of patients with ADHF had hypertension, 60% had CAD, and 40% had diabetes [16].

Patients with AF and ADHF had more frequent rehospitalization rates and longer hospital stays than patients without AF [21-23]. This indicates that apart from structural abnormalities, there are several other risk factors that significantly contribute to the development of AF and adverse clinical outcomes.

Limitations

Our study was limited to a single-center study of one tertiary care hospital due to a shortage of time. The study examined a limited number of risk factors due to limited funding. Future research would benefit from multicenter research with a broader range of risk factors studied.

## Conclusions

We conducted this study to determine the prevalence of AF among patients with ADHF in Pakistan. AF occurs in a significant amount of patients with ADHF, and the risk factors associated with AF in these patients include hypertension, history of MI, diabetes, and COPD. These risk factors with both AF and ADHF can lead to poor patient outcomes. Healthcare professionals should screen patients with ADHF for AF, especially in the presence of common risk factors.
